# Don’t judge the myocardium by its cover: The incremental value of cardiac magnetic resonance imaging in left ventricular hypertrophy

**DOI:** 10.1007/s12471-017-1069-x

**Published:** 2017-12-19

**Authors:** R. Y. Parbhudayal, C. P. Allaart, R. B. van Loon, L. J. Meijboom, A. C. van Rossum, R. Nijveldt

**Affiliations:** 1 Department of Cardiology, VU University Medical Center, Amsterdam, The Netherlands; 2 Department of Radiology, VU University Medical Center, Amsterdam, The Netherlands

A 51-year-old female presenting with fatigue demonstrated cardiac enlargement on her chest x‑ray. The electrocardiogram was normal (Fig. 1). However, echocardiography suggested asymmetrical hypertrophic cardiomyopathy. Cardiac magnetic resonance (CMR) imaging was performed for further evaluation. The cine images showed a maximum wall thickness of 28 mm at the mid anterolateral segment (Fig. 2a; Supplementary movie 1). Due to the atypical location, additional sequences were acquired. T2-weighted (water) imaging showed a high signal intensity (Fig. 2b), whereas T1-weighted imaging was normal. During first pass perfusion imaging, the hypertrophied myocardium showed instant contrast enhancement (Fig. 2c; Supplementary movie 2), with homogeneous hyperenhancement at late gadolinium-enhancement (Fig. 2d), suggesting cardiac haemangioma. This was confirmed by coronary angiography demonstrating a tumorous blush (Supplementary movie 3). Extra-cardiac tumours were excluded with a fluorine 18 fluorodeoxyglucose positron emission tomography (FDG-PET) scan. Primary cardiac haemangiomas are rare, with an incidence of 2% of all primary cardiac tumours [1]. Most cases present with arrhythmia, which did not occur in our patient. Depending on their location, haemangiomas could interfere with the conduction system. In our patient, however, the ECG was completely normal. Cardiac haemangiomas may regress or proliferate and surgical resection should be considered in case of interference in outflow tract or conduction [2]. Although, echocardiography remains the first technique to evaluate left ventricular hypertrophy, CMR imaging is indispensable for further characterisation and might even identify other diagnoses.

**Fig. 1:**
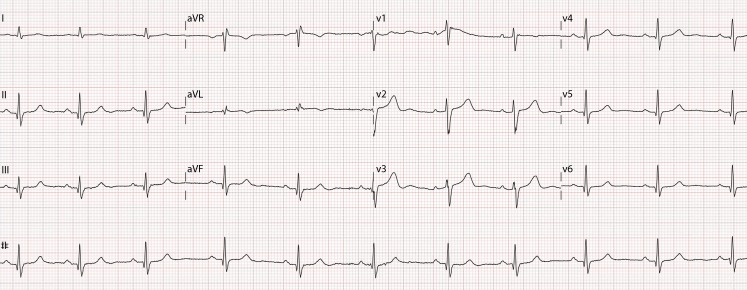
The normal electrocardiogram

**Fig. 2:**
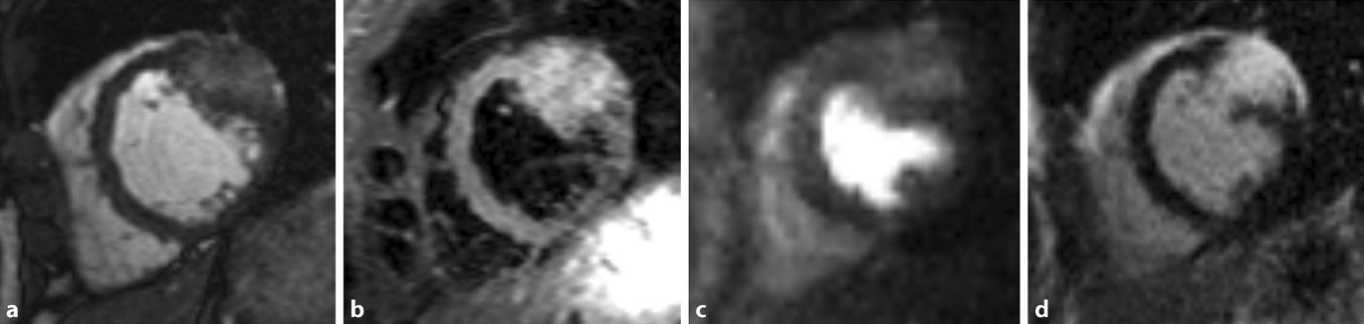
Cardiac magnetic resonance images showing a cardiac haemangioma in the short-axis view in the cine (**a**), T2-weighted (water) (**b**), first pass perfusion (**c**) and late gadolinium-enhancement (**d**) image

## Caption Electronic Supplementary Material

Video 1 = Movie Cine Sax LV

Video 2 = Movie First pass perfusion Sax LV

Video 3 = Coronary angiography
